# Aclarubicin Reduces the Nuclear Mobility of Human DNA Topoisomerase IIβ

**DOI:** 10.3390/ijms251910681

**Published:** 2024-10-03

**Authors:** Keiko Morotomi-Yano, Ken-ichi Yano

**Affiliations:** 1 Institute of Industrial Nanomaterials, Kumamoto University, Kumamoto 860-8555, Japan; 2 Faculty of Advanced Science and Technology, Kumamoto University, Kumamoto 860-8555, Japan

**Keywords:** aclarubicin, DNA topoisomerase II, TOP2 inhibitor, live cell imaging

## Abstract

DNA topoisomerase II (TOP2) is an enzyme that resolves DNA topological problems arising in various nuclear processes, such as transcription. Aclarubicin, a member of the anthracyclines, is known to prevent the association of TOP2 with DNA, inhibiting the early step of TOP2 catalytic reactions. During our research on the subnuclear distribution of human TOP2B, we found that aclarubicin affects the mobility of TOP2B in the nucleus. FRAP analysis demonstrated that aclarubicin decreased the nuclear mobility of EGFP-tagged TOP2B in a concentration-dependent manner. Aclarubicin exerted its inhibitory effects independently of TOP2B enzymatic activities: TOP2B mutants defective for either ATPase or topoisomerase activity also exhibited reduced nuclear mobility in the presence of aclarubicin. Immunofluorescence analysis showed that aclarubicin antagonized the induction of DNA damage by etoposide. Although the prevention of the TOP2-DNA association is generally considered a primary action of aclarubicin in TOP2 inhibition, our findings highlight a previously unanticipated effect of aclarubicin on TOP2B in the cellular environment.

## 1. Introduction

When a DNA double helix is unwound for strand separation, torsional strain is produced, which leads to various topological problems for DNA [1,2]. An ATP-dependent enzyme called DNA topoisomerase II (TOP2) solves the topological problems caused by DNA unwinding [1,2,3]. TOP2 plays a critical role in ensuring the execution of various nuclear processes accompanied by DNA unwinding, such as transcription, DNA replication, and chromosome segregation [1,2,3]. Human cells have two TOP2, namely TOP2A and TOP2B [4,5]. TOP2A is expressed in a cell-cycle-dependent manner and is present in proliferating cells [6,7]. TOP2A plays an essential role in cell division, particularly chromosome segregation [7,8]. TOP2B exists throughout the cell cycle and functions in both dividing and nondividing cells [7]. TOP2B is involved in the control of transcription [9,10,11] and the chromatin architecture [12,13,14]. Although TOP2A and B have different roles in cell physiology [2,3], they are structurally similar: They consist of the N-terminal ATPase and the central catalytic domains [1,15]. They form homo dimers [1] and exhibit similar enzymatic properties in vitro [16,17].

To relieve the torsional strain of DNA, TOP2 functions through multiple steps that form a so-called catalytic cycle [18,19]. TOP2 starts its catalytic cycle by attaching to DNA segments. Afterward, TOP2 binds to ATP and undergoes conformational changes. TOP2 and DNA form a TOP2-DNA cleavage complex, and TOP2 introduces a DNA double-strand break (DSB) in one segment of DNA. A DSB serves as a gate and allows another DNA segment to pass through it, relieving torsional strain. TOP2 religates the DSBs to restore the integrity of DNA. Finally, TOP2 hydrolyzes ATP and dissociates from DNA strands, returning to the initial step of the catalytic cycle [18,19].

Various compounds have been developed to impede the TOP2 catalytic cycle [19]. For example, etoposide stabilizes the TOP2-DNA cleavage complex and prevents the religation of DSBs [20]. In a cell, stabilized TOP2-DNA cleavage complexes are readily converted into DSBs, which can trigger cell death, particularly in rapidly dividing cells, such as cancer cells. Etoposide is referred to as a TOP2 poison and is regarded as an effective chemotherapy agent [20]. Another class of drugs targeting the TOP2 catalytic cycle is TOP2 catalytic inhibitors, such as ICRF-187. ICRF-187 inhibits the ATPase activity of TOP2 and traps a TOP2 homodimer in a closed conformation around DNA, which is called a closed clamp [21,22]. Thus, ICRF-187 tethers TOP2 to DNA strands in the late step of the catalytic cycle [1,2].

Aclarubicin is a member of the anthracyclines and was originally identified as an anti-tumor antibiotic [23]. Although both the TOP2 catalytic inhibitors and TOP2 poisons act on DNA-bound TOP2, aclarubicin exerts its inhibitory action differently [19]. Aclarubicin intercalates into DNA double helices and induces structural distortions in DNA. Gel shift assays using a purified TOP2 protein and a radiolabeled DNA probe have demonstrated that aclarubicin completely interferes with the association between TOP2 and DNA [24]. Thus, the primary inhibitory mechanism of aclarubicin on TOP2 was proposed to prevent the association of TOP2 with DNA in the early step of the catalytic cycle [19,25]. Consistent with this view, cell-based assays have shown that aclarubicin antagonizes the cellular toxicity of TOP2 poisons, such as etoposide [26,27] and doxorubicin [28].

In addition to its TOP2 inhibition, aclarubicin exhibits multiple biological actions. First, aclarubicin causes increased reactive oxygen species levels and mitochondrial dysfunction [29,30]. Second, aclarubicin alters the accessibility of RNA polymerase II to promoter regions, leading to enhanced transcriptional elongation [31]. Next, Western blot analysis of nuclear proteins showed aclarubicin-mediated degradation of RNA polymerase II [32] and increases in the chromatin-bound fractions of the histone chaperone FACT [33] and TOP2A and TOP2B [32]. Finally, aclarubicin at relatively high concentrations induces histone eviction [34,35].

Human TOP2B is highly mobile in the nucleus and localizes in both the nucleoplasm and nucleoli [36,37,38]. Previous studies have demonstrated that the subnuclear distribution of TOP2B alters in response to cellular ATP levels. When intracellular ATP is depleted by inhibiting the glycolytic and mitochondrial pathways for ATP production, TOP2B quickly accumulates in the nucleoli [37,38]. We were intrigued by this phenomenon and searched for drugs that could affect it. In the course of our research, we found that aclarubicin reduces the mobility of TOP2B. Because aclarubicin is known to interfere with the TOP2-DNA association [24], the observed reduction in the nuclear mobility of TOP2B suggests that aclarubicin augments the association of TOP2B with some nuclear components other than DNA. Disruptions of TOP2B catalytic sites did not alleviate the aclarubicin-mediated reduction in TOP2B mobility, supporting the idea that TOP2B associates with non-DNA components in the presence of aclarubicin. Our observations suggest that the inhibitory action of aclarubicin on TOP2 in the cellular environment is more complicated than currently recognized.

## 2. Results

### 2.1. Aclarubicin Inhibits Subnuclear Translocation of TOP2B in ATP-Depleted Cells

**Figure 1:**
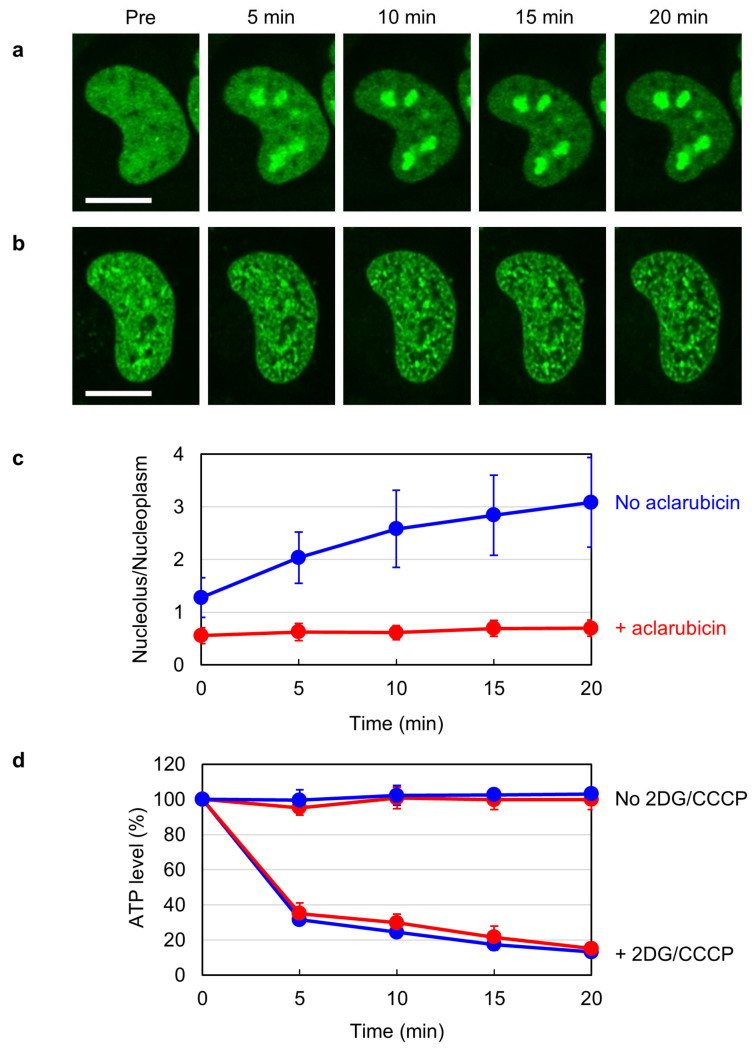
Aclarubicin inhibits the nucleolar translocation of EGFP-TOP2B in ATP-depleted cells. (**a**) Representative images of EGFP-TOP2B in an ATP-depleted cell. The EGFP-TOP2B expression plasmid was transiently transfected into HeLa cells. Cells were treated with 25 mM 2DG and 10 µM CCCP, and fluorescent images were captured at the indicated periods. Bar: 10 µm. (**b**) Representative images of EGFP-TOP2B in an aclarubicin-treated ATP-depleted cell. Cells were treated with 1 µM aclarubicin for 60 min. 2DG and CCCP were added after aclarubicin pretreatment. Bar: 10 µm. (**c**) Quantification of the nucleolus/nucleoplasm ratio of EGFP-TOP2B. Fluorescence in the nucleolus and nucleoplasm was quantified. Average values of the nucleolus/nucleoplasm ratio and SD were calculated (*n* = 10). (**d**) Measurement of intracellular ATP levels. Cells were treated with (red) or without (blue) 1 µM aclarubicin for 60 min and subsequently treated with or without 25 mM 2DG and 10 µM CCCP. Experiments were repeated five times, and average values with SD were calculated.

Previous studies have shown that the distribution of TOP2B dynamically changes in response to cellular ATP levels [37,38]. When HeLa cells expressing EGFP-tagged TOP2B (EGFP-TOP2B) were depleted of ATP via the addition of 2-deoxyglucose (2DG) and CCCP, EGFP-TOP2B rapidly accumulated in the nucleoli (Figure 1a) [38]. We were interested in this phenomenon and tried to understand the underlying mechanism. In the course of our study, we observed that pretreatment of cells with 1 µM aclarubicin suppressed the accumulation of EGFP-TOP2B in the nucleoli of ATP-depleted cells (Figure 1b). Quantification of the fluorescence of EGFP-TOP2B in the nucleoplasm and the nucleoli confirmed the inhibitory effect of aclarubicin on the nucleolar accumulation of TOP2B (Figure 1c). We verified that aclarubicin affected neither normal cellular ATP levels nor ATP depletion by 2DG/CCCP treatment (Figure 1d).

**Figure 2:**
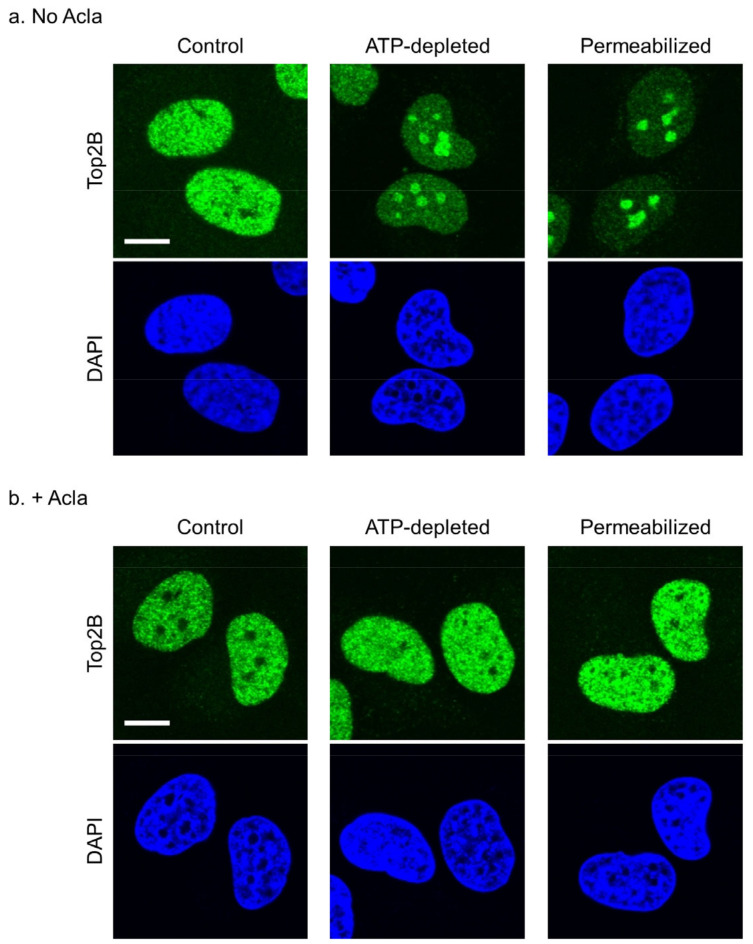
Aclarubicin suppresses the nucleolar translocation of endogenous TOP2B. Immunostaining experiments were repeated 5 times, and representative images from the final experiment are shown. (**a**) Immunostaining of endogenous TOP2B. HeLa cells were ATP-depleted with 25 mM 2DG and 10 µM CCCP for 20 min or membrane-permeabilized with 0.4% Triton X-100. Cells were fixed and immunostained with anti-TOP2B antibody. The nuclei were costained with DAPI. Bar: 10 µm. (**b**) Immunostaining of endogenous TOP2B in aclarubicin-treated cells. Cells were pretreated with 1 µM aclarubicin for 60 min and subsequently subjected to ATP-depletion or membrane-permeabilization.

Next, we examined the effect of aclarubicin on the subnuclear distribution of endogenous TOP2B using immunofluorescence microscopy. In ATP-depleted cells, endogenous TOP2B accumulated in the nucleoli (Figure 2a). Membrane permeabilization is known to induce the nucleolar accumulation of TOP2B (Figure 2a), presumably because of the leakage of cellular ATP [37]. When cells were treated with aclarubicin, neither ATP depletion nor membrane permeabilization caused the nucleolar accumulation of endogenous TOP2B (Figure 2b). Taken together, these results show that aclarubicin inhibits the nucleolar translocation of TOP2B.

### 2.2. Aclarubicin Reduces the Nuclear Mobility of TOP2B2.1

Because TOP2B is highly mobile in a living cell [36,37,38], we next carried out FRAP analysis to inquire whether aclarubicin interferes with TOP2B nucleolar translocation by affecting the mobility of TOP2B. First, we photobleached green fluorescence in a small defined area in the nucleus of an EGFP-TOP2B expressing cell. We then monitored the recovery of fluorescence in the bleached area over time. The speed of fluorescence recovery is generally viewed as reflecting the mobility of an EGFP-tagged protein: fast and slow recovery rates indicate high and low protein mobilities, respectively.

**Figure 3:**
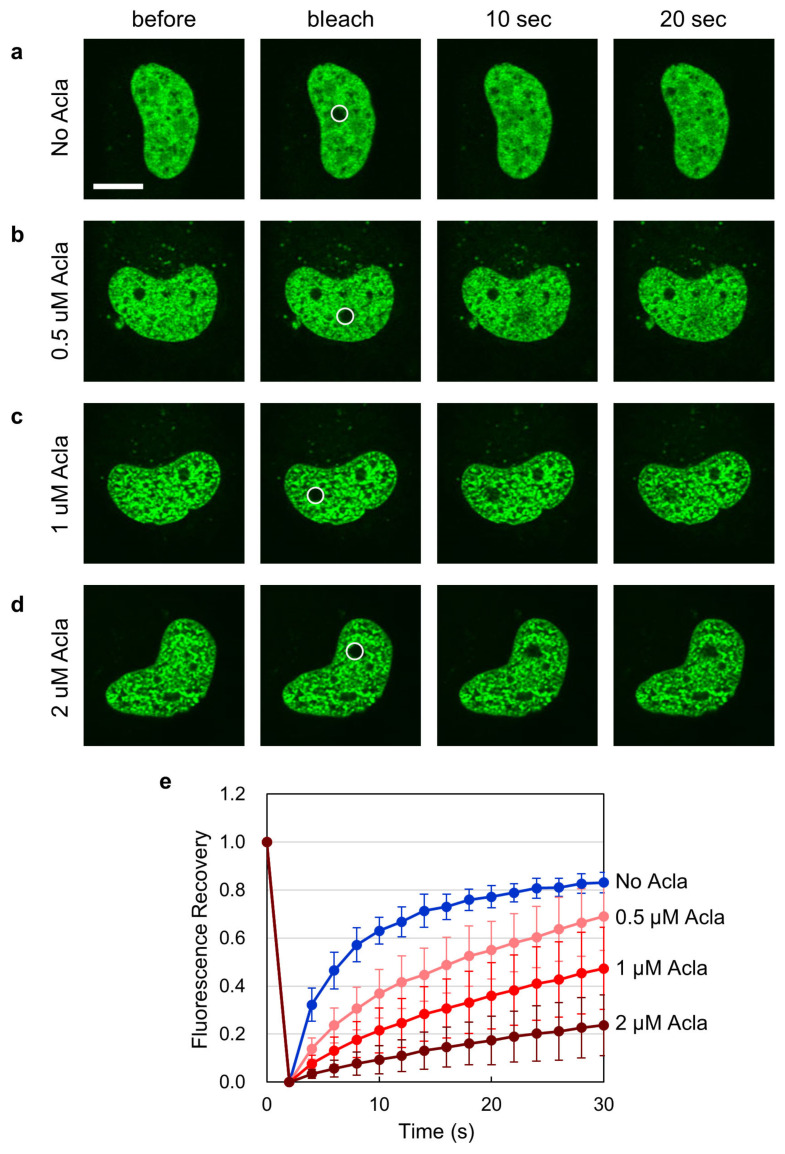
Aclarubicin reduces the nuclear mobility of EGFP-TOP2B. (**a**–**d**) Representative images of FRAP analysis of EGFP-TOP2B in the presence of aclarubicin. HeLa cells expressing EGFP-TOP2B were treated with 0, 0.5, 1, and 2 µM aclarubicin for 1 h. Photobleaching was carried out in a small area of the nucleus (shown with a circle), and fluorescence recovery was monitored. Bar: 10 µm. (**e**) Quantitative analysis of fluorescence recovery of EGFP-TOP2B in the presence of aclarubicin. Fluorescence images were captured at 2 s intervals. Photobleaching was conducted at 2 s. Average values of relative fluorescence and SD were calculated from 10 cells. The t1/2 of fluorescence recovery was calculated to be 3.68  ±  0.79 s (0 µM), 10.14  ±  3.15 s (0.5 µM), 16.65  ±  5.52 s (1 µM), and 26.14  ±  13.01 s (2 µM).

As shown in Figure 3a, without aclarubicin treatment, the fluorescence in the bleached area was rapidly recovered, indicating the high mobility of EGFP-TOP2B, as reported previously [38]. When cells were treated with increasing concentrations of aclarubicin, the fluorescence recovery was reduced in a concentration-dependent manner (Figure 3b–d). Quantification of the fluorescence recovery confirmed that aclarubicin reduced the nuclear mobility of EGFP-TOP2B (Figure 3e). These results suggest that the effect of aclarubicin on TOP2B mobility can account for the inhibition of TOP2B nucleolar translocation. We speculate that aclarubicin may enhance the association of TOP2B with less mobile nuclear components, such as chromatin.

### 2.3. Inhibitory Effect of Aclarubicin on TOP2B Mobility Is Independent of TOP2B Enzymatic Activities

**Figure 4:**
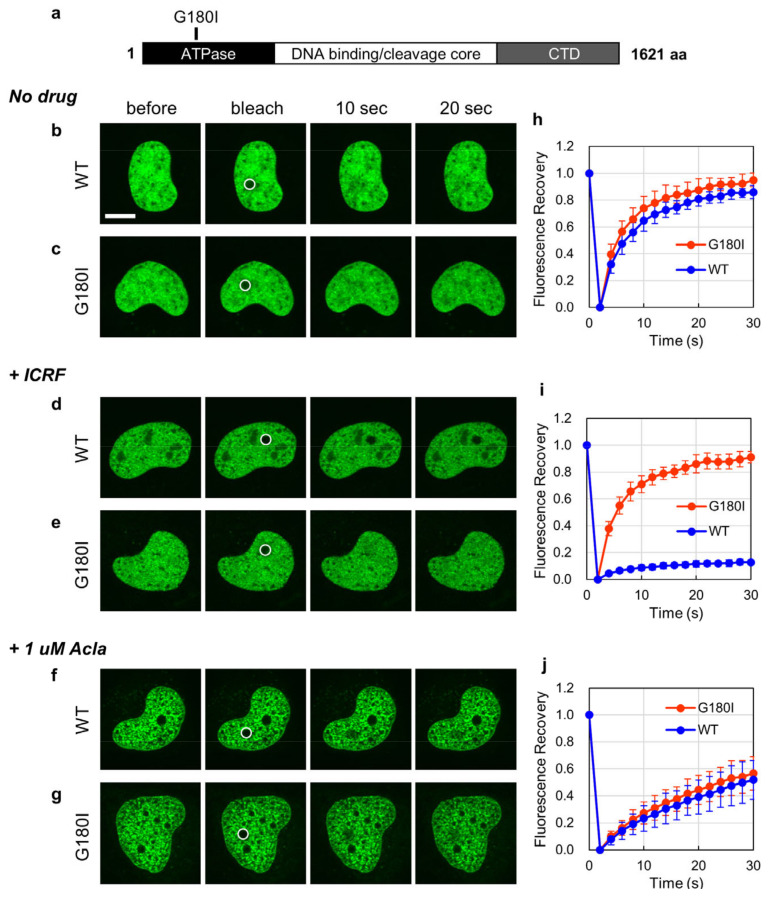
Aclarubicin reduces the nuclear mobility of ICRF-insensitive EGFP-TOP2B. (**a**) Structure of human TOP2B. TOP2B with a G180I substitution in its ATPase domain is insensitive to ICRF-187. (**b**–**g**) Representative images of FRAP analysis of EGFP-TOP2B WT and G180I. FRAP analysis was performed without drug pretreatment (**b**,**c**) and after a 1 h pretreatment with 20 µM ICRF-187 (**d**,**e**) or 1 µM aclarubicin (**f**,**g**). Bleached areas are shown with circles. Bar: 10 µm. (**h**–**j**) Quantitative analysis of fluorescence recovery of EGFP-TOP2B WT and G180I. Average values and SD were calculated from 10 cells.

ICRF-187 is a TOP2 catalytic inhibitor that halts an ATP-bound form of TOP2B on DNA as a closed clamp [21,22], reducing the nuclear mobility of TOP2B [39]. This action of ICRF-187 can be abrogated by the substitution of glycine 180 with isoleucine (G180I) in the ATPase domain of TOP2B [37,40] (Figure 4a). We next attempted to compare the effects of ICRF-187 and aclarubicin on the nuclear mobility of wild-type (WT) and G180I-carrying EGFP-TOP2B. Without ICRF-187 or aclarubicin, both EGFP-TOP2B WT and G180I were highly mobile in FRAP analysis (Figure 4b,c,h). The treatment of cells with ICRF-187 significantly reduced the nuclear mobility of TOP2B WT but not G180I (Figure 4d,e,i), confirming that the action of ICRF-187 requires the functional ATPase of TOP2B. On the other hand, aclarubicin reduced the mobilities of TOP2B WT and G180I to a similar extent (Figure 4f,g,j). Together, unlike ICRF-187, the effect of aclarubicin on TOP2B mobility is independent of the ATPase activity of TOP2B.

**Figure 5:**
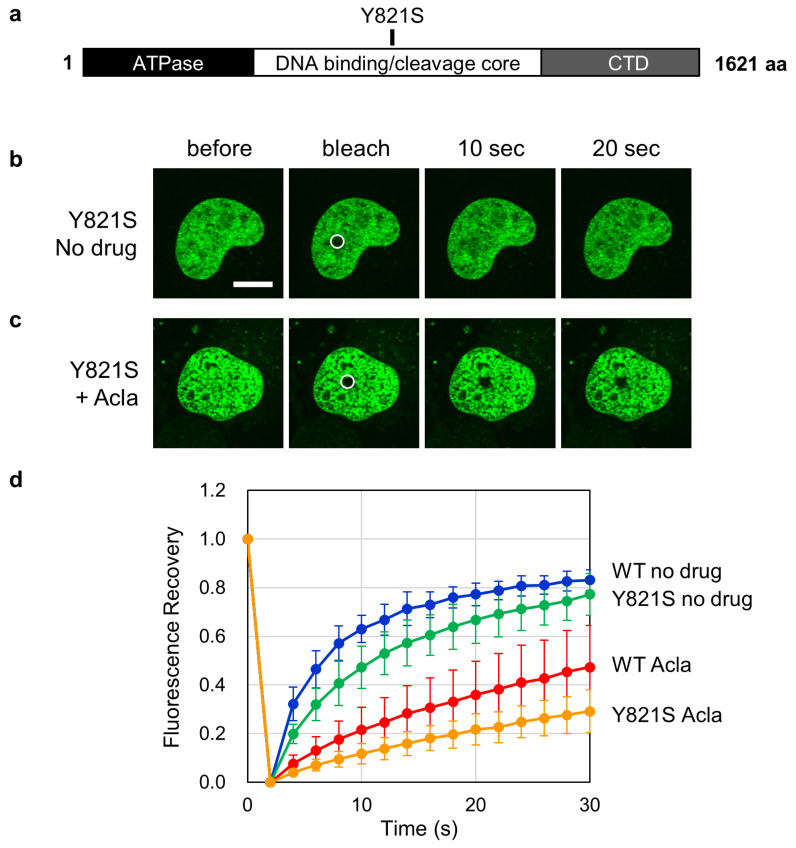
Aclarubicin reduces the nuclear mobility of catalytically inactive EGFP-TOP2B. (**a**) Structure of human TOP2B. TOP2B with a Y821S substitution in its DNA-binding/cleavage core domain is catalytically inactive. (**b**,**c**) Representative images of FRAP analysis of EGFP-TOP2B Y821S. FRAP analysis was performed without (**b**) and with a 1 h pretreatment with 1 µM aclarubicin (**c**). Bleached areas are shown with circles. Bar: 10 µm. (**d**) Quantitative analysis of fluorescence recovery of EGFP-TOP2B WT and Y821S. Average values and SD for EGFP-TOP2B Y821S were calculated from 10 cells. The data for EGFP-TOP2B WT with/without 1 µM aclarubicin pretreatment are the same as those in Figure 3e.

We next asked whether the topoisomerase activity is involved in the effect of aclarubicin on TOP2B mobility. A previous study has shown that the substitution of tyrosine 821 with serine (Y821S, Figure 5a) abolishes the topoisomerase activity of TOP2B [37,41]. By means of FRAP analysis, we observed that EGFP-TOP2B Y821S was mobile in the absence of aclarubicin (Figure 5b). Quantification of fluorescence recovery exhibited that the mobility of EGFP-TOP2B Y821S was slightly lower than that of WT (Figure 5d). When cells were treated with aclarubicin, the mobility of EGFP-TOP2B Y821S was significantly reduced to a lower level than WT. This result indicates that the disruption of topoisomerase activity did not relieve the inhibitory effect of aclarubicin on TOP2B mobility (Figure 5b,d). Taken together, the observations on G180I and Y821S demonstrate that the effect of aclarubicin on TOP2B mobility does not require the enzymatic activities of TOP2B. The dispensability of the enzymatic activities implies that aclarubicin affects a DNA-unbound form of TOP2B in the cell.

### 2.4. Aclarubicin Reduces Etoposide-Induced DNA Damage

**Figure 6:**
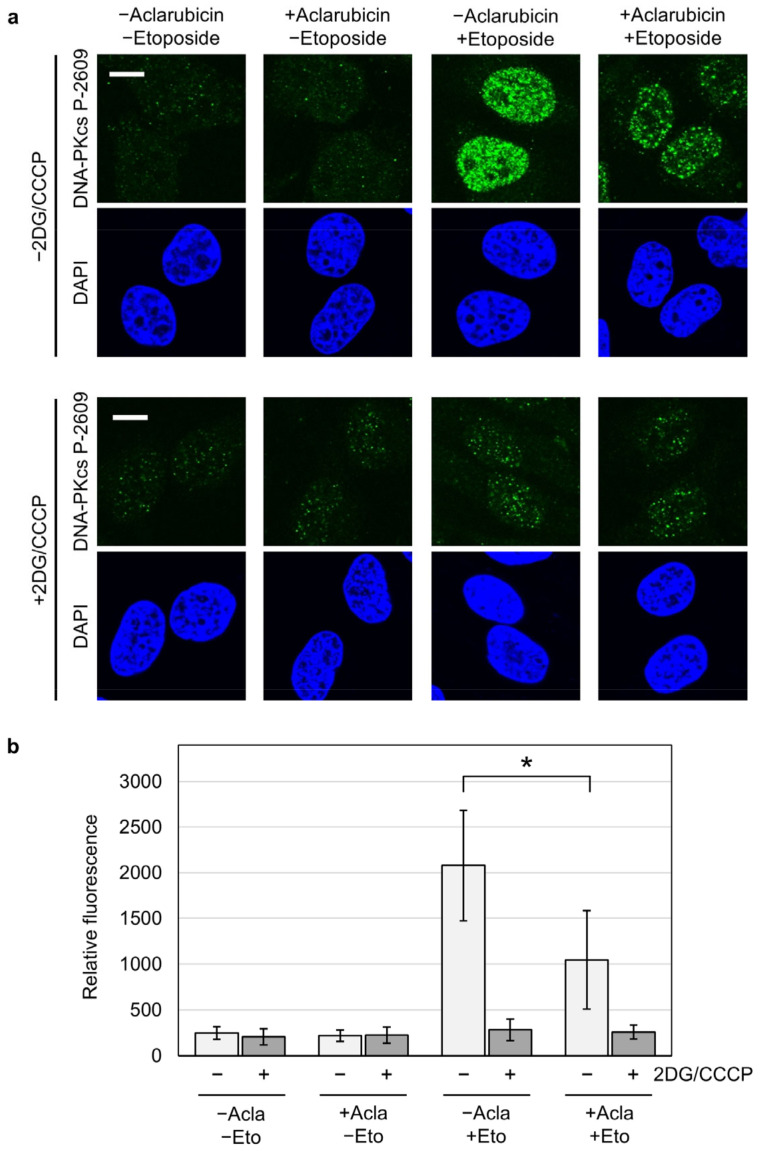
Aclarubicin reduces etoposide-induced DNA damage. (**a**) Representative images of immunostaining for the phosphorylated threonine 2609 of DNA-PKcs. HeLa cells were treated with a combination of 1 µM aclarubicin and 25 µM etoposide for 1 h with or without ATP depletion by 25 mM 2DG and 10 µM CCCP. Cells were fixed and immunostained for the phosphorylated threonine 2609 of DNA-PKcs. (**b**) Quantification of fluorescence of the phosphorylated threonine 2609 of DNA-PKcs. Average values and SD were calculated from 50 cells. *: Statistically significant (*p* = 3.4 × 10^−11^).

A previous study showed that aclarubicin prevents the association of a purified TOP2 protein and a DNA probe in vitro [24]. Consistent with the in vitro data, cell-based assays demonstrated that aclarubicin antagonizes the cytotoxicity of etoposide [26,27]. Next, we sought to confirm the antagonizing effect of aclarubicin on etoposide-induced DSB formation. We treated HeLa cells with a combination of aclarubicin and etoposide for 1 h. Then, we subjected the cells to fixation followed by immunofluorescence of a DSB marker (phosphorylated threonine 2609 of DNA-PKcs) [42]. As shown in Figure 6a, aclarubicin alone did not increase DSB formation, which agrees with previous studies [34,43]. Etoposide treatment caused the strong induction of DSBs, as anticipated (Figure 6a). When cells were treated with aclarubicin and etoposide simultaneously, the induction of DSBs was significantly reduced (Figure 6a). Through the quantification of the fluorescence signals, we confirmed that aclarubicin attenuated the effect of etoposide on DSB induction (Figure 6b). These results confirm that aclarubicin antagonizes the DSB-inducing action of etoposide. When the experiment was performed under ATP depletion, the etoposide-induced phosphorylation of DNA-PKcs was significantly reduced, presumably because ATP depletion prevents both protein phosphorylation and the action of etoposide (Figure 6).

## 3. Discussion

TOP2B is known to be highly mobile in the nucleus, and its subnuclear distribution alters in response to cellular ATP levels [36,37,38]. A low ATP state triggers the translocation of TOP2B from the nucleoplasm to the nucleoli, which is proposed to be a novel proteostatic mechanism to prevent unfavorable actions of TOP2B via nucleolar sequestration [38]. In the course of research on the subnuclear translocation of TOP2B, we found that aclarubicin markedly affects the nuclear mobility of TOP2B. FRAP analysis demonstrated that aclarubicin reduces TOP2B mobility in a dose-dependent manner. A previous study using a purified TOP2 protein demonstrated that aclarubicin prevents the association of TOP2 with DNA in vitro [24]. We thus speculate that the reduced nuclear mobility of TOP2B by aclarubicin could be ascribed to the augmented association of TOP2B with a non-DNA component of chromatin. Accordingly, the enzymatic activities of TOP2B were indispensable for the effect of aclarubicin, which is in contrast to the case of ICRF-187: ICRF-187 halts TOP2 as a closed clamp on DNA [21,22], and its action requires the functional ATPase of TOP2 [37,40]. Furthermore, the disruption of topoisomerase activity by the Y821S substitution did not relieve the reduced mobility of TOP2B by aclarubicin. These observations imply that a previously unanticipated effect of aclarubicin on TOP2B exists in the cellular environment.

Although this study reveals a novel effect of aclarubicin on the nuclear mobility of TOP2B, it raises new questions for further investigation. The first question is the mechanism for the reduction in TOP2B mobility by aclarubicin. As mentioned above, we suppose that aclarubicin augments the association of TOP2B with a non-DNA component of chromatin. Proteomics analysis has identified multiple TOP2B-interacting proteins, including CCCTC-binding factor (CTCF) and the subunits of cohesin [12,44]. CTCF and cohesin colocalize at many sites across the human genome and work in coordination to control chromatin structure [45,46]. In addition to evidence from proteomics, genome-wide analysis of TOP2B binding sites has demonstrated that TOP2B is frequently localized at overlapping or adjacent positions to the CTCF/cohesin-binding sites on the genome [12,13,14,44,47]. Interestingly, the association of CTCF and cohesin with chromatin is much more stable than that of TOP2B. The half-time of fluorescence recovery of EGFP-TOP2B was estimated to be a few seconds (Figure 3e and Figure 4h), indicating the high mobility of TOP2B. On the other hand, the residence times of CTCF and cohesin in chromatin were reported to be 1 min and 22 min, respectively [48]. If aclarubicin stabilizes the association of TOP2B with either CTCF or cohesion, TOP2B mobility decreases, as observed in this study. Another possible mechanism for the reduced TOP2B mobility may be the interaction of TOP2B with stable chromatin components that are not recognized as TOP2B-interacting partners. The comparison of TOP2B-interacting factors in the presence and absence of aclarubicin will provide clues to understanding the mechanism for the reduced TOP2B mobility caused by aclarubicin.

Another question to be explored in the future is the impact of its reduced mobility on the physiological role of TOP2B. Many proteins, including TOP2B, are highly mobile in the nucleus and exhibit rapid association and dissociation with their target sites [49,50,51]. High protein mobility in the nucleus is generally beneficial for cellular physiology [51,52,53]. First, high mobility can facilitate targeting a protein to its specific binding sites. Second, the rapid association with and dissociation of a protein from its target site enables the efficient redistribution of a protein to different sites in response to cellular signaling and environmental changes. Thus, aclarubicin may restrain the timely distribution to target sites and regulatory plasticity of TOP2B in a cell. Aclarubicin is well known to interfere with the TOP2-DNA association in vitro, and future research will further advance our understanding of how aclarubicin affects the function of TOP2B in the cellular environment.

## 4. Materials and Methods

### 4.1. Reagents, Antibodies, and Plasmid

Aclarubicin was purchased from Focus Biomolecules (Plymouth Meeting, PA, USA). 2-deoxy-glucose (2DG) and etoposide were obtained from FUJIFILM Wako Pure Chemical (Osaka, Japan). ICRF-187 and carbonyl cyanide m-chlorophenylhydrazone (CCCP) were purchased from Cayman Chemical (Ann Arbor, MI, USA) and Sigma-Aldrich (St. Louis, MO, USA), respectively. Anti-TOP2B mouse monoclonal antibody was obtained from BD Biosciences (#611492, Franklin Lakes, NJ, USA). Goat anti-mouse IgG-Alexa Fluor488 antibody was from Thermo Fisher Scientific (A11029, Waltham, MA, USA). The expression plasmids for EGFP-TOP2B WT, G180I, and Y821S were described previously [54].

### 4.2. Cell Culture and Transfection

HeLa cells were obtained from RIKEN BioResource Research Center (Wako, Japan). Cells were grown in α-modified minimum essential medium (αMEM, FUJIFILM Wako Pure Chemical, Osaka, Japan) supplemented with 10% fetal bovine serum (Corning, NY, USA), 100 µg/mL streptomycin, and 100 units/mL penicillin (FUJIFILM Wako Pure Chemical, Osaka, Japan). Cells were cultured under standard conditions at 37 °C in a humidified incubator containing 5% CO_2_.

For plasmid transfection, cells (1 × 10^5^) were plated in a 35 mm glass-bottomed dish (Matsunami, Osaka, Japan). After 24 h of incubation at 37 °C, a plasmid (300 ng) was transfected using a FuGENE HD reagent (Promega, Madison, WI, USA) according to the manufacturer’s instructions. At 30 h after transfection, cells were used for live cell imaging and FRAP analysis.

### 4.3. ATP Measurement

Cells were plated in a 96-well plate (5 × 103 cells/well). After 24 h of incubation at 37 °C, cells were pretreated with or without 1 µM aclarubicin for 1 h. Cells were subsequently treated with 25 mM 2DG and 10 µM CCCP for periods indicated in Figure 1d. Cell lysis and ATP measurement were performed using a CellTiter-Glo luminescent cell viability assay kit (Promega, Madison, WI, USA) according to the manufacturer’s instructions. Luminescence was measured using a 2030 ARVO X multilabel reader (Perkin Elmer, Shelton, CT, USA).

### 4.4. Fluorescence Microscopy and Live Cell Imaging

Fluorescence microscopy and live cell imaging were carried out as described previously [39]. Briefly, an FV1200-IX83 laser scanning confocal microscope with an oil-immersed 60× objective (Olympus, Tokyo, Japan) was used for fluorescence microscopy. For live cell imaging, cells plated in a glass-bottomed 35 mm dish were placed on a stage top incubator (Tokai Hit, Fujinomiya, Japan) that maintained a humidified atmosphere of 5% CO_2_ at 37 °C. Images were captured and analyzed using FLUOVIEW software (Version 4.1, Olympus, Osaka, Japan).

### 4.5. Immunofluorescence Staining

Cells were fixed with 4% paraformaldehyde dissolved in Dulbecco’s phosphate-buffered saline (D-PBS) for 15 min at 4 °C. Fixed cells were washed with D-PBS three times and subsequently permeabilized with 0.4% Triton X-100 in D-PBS at room temperature for 5 min. Fixed and permeabilized cells were blocked with 1% bovine serum albumin in D-PBS for 15 min and reacted with mouse anti-TOP2B monoclonal antibody at 4 °C for 4 h. After washing with D-PBS, cells were incubated with goat anti-mouse IgG-Alexa Fluor488 antibody at 4 °C for 1 h. Cells were mounted in a Vectashield mounting medium with DAPI (Vector Laboratories, Newark, CA, USA).

For membrane permeabilization prior to fixation, detergent treatment was performed according to previously described procedures [55] with slight modifications. Cells were incubated twice for 3 min at room temperature with cytoskeleton buffer (CSK) containing 10 mM Pipes (pH 7.0), 100 mM NaCl, 300 mM sucrose, 3 mM MgCl2, and 0.4% Triton X-100. After washing with D-PBS, cells were fixed with 4% PFA and subsequently subjected to immunofluorescence staining as described above.

### 4.6. FRAP Analysis

FRAP analysis was performed as reported previously [39]. Briefly, transfection and live cell imaging were carried out as described above. A spot in the nucleoplasm of a transfected cell was photobleached with a 473 nm laser at 35% output for 1 s. Before and after photobleaching, fluorescent images were captured at 2 s intervals. The intensities of fluorescence of photobleached and unbleached areas in the same cell were quantified using FLUOVIEW software (Version 4.1, Olympus, Tokyo, Japan). The intensities of fluorescence before and immediately after photobleaching were set to 1 and 0, respectively. Fluorescence recovery was expressed as a ratio of fluorescence before and after photobleaching. FRAP curve fitting by means of non-linear regression was carried our using the Stowers ImageJ plugins (https://research.stowers.org/imagejplugins/ImageJ_tutorial2.html. Accessed in 2023 and 2024.) in Fiji (ImageJ version 1.53t) [56] according to the procedures described previously [57]. The normalized fluorescence recovery curves were fit with an exponential recovery function using the ImageJ plugin ‘batch FRAP fit jru v1’ in the Stowers ImageJ plugins (https://research.stowers.org/imagejplugins/ImageJ_tutorial2.html. Accessed in 2023 and 2024). The half-time of fluorescence recovery (t1/2) was calculated for each curve.

### 4.7. Statistical Analysis

Welch’s *t*-test was used for statistical analysis. The number of experiments and *p*-values are indicated in the figure legends.

## Data Availability

The data that support the findings of this study are available from the corresponding authors upon reasonable request.
